# Knowledge, attitudes, and practices regarding lumbar disc herniation among diagnosed patients

**DOI:** 10.3389/fpubh.2025.1583361

**Published:** 2025-06-26

**Authors:** Ruijun Xu, Mingliang Ning, Ronghua Yu, Mingchen Yu, Jiangming Yu, Haojie Chen

**Affiliations:** ^1^Department of Orthopedics, Tongren Hospital, Shanghai Jiao Tong University School of Medicine, Shanghai, China; ^2^Department of Orthopedics, Changzhou Geriatric Hospital Affiliated to Soochow University, Changzhou, China

**Keywords:** lumbar disc herniation, knowledge, attitudes, practice, cross-sectional study, structural equation modeling, KAP

## Abstract

**Introduction:**

This study aimed to investigate the knowledge, attitudes, and practices (KAP) of patients with lumbar disc herniation (LDH) regarding their condition.

**Methods:**

A cross-sectional study was conducted between June and September 2024 at Tongren Hospital in Shanghai and the Seventh People’s Hospital of Changzhou, targeting patients diagnosed with LDH. Demographic information and KAP scores were collected and evaluated through a structured questionnaire. The Oswestry Disability Index (ODI) was used to assess the level of disability experienced by patients in daily activities.

**Results:**

A total of 395 valid questionnaires were collected, yielding an effective rate of 84.40%. Of the respondents, 205 (51.90%) were female, 201 (50.89%) had undergone surgical treatment. The mean ± SD scores for knowledge, attitude, practice, and ODI were 14.91 ± 5.07 (possible range: 0–22), 39.26 ± 3.78 (possible range: 10–50), 39.48 ± 6.70 (possible range: 11–55), and 12.92 ± 9.60 (possible range: 0–50), respectively. The Structural Equation Model indicated that knowledge had a direct effect on both attitude (*β* = 0.458, *p* = 0.006) and practice (*β* = 0.214, *p* = 0.002), while attitude directly affected practice (*β* = 0.323, *p* = 0.008). Knowledge also indirectly influenced practice via attitude (*β* = 0.148, *p* = 0.006).

**Conclusion:**

Patients with LDH demonstrated inadequate knowledge but generally positive attitudes and proactive practices regarding their condition, along with a moderate level of disability related to low back pain. Improving patient education, particularly focusing on enhancing knowledge, may foster more positive attitudes and better self-management practices, potentially reducing disability.

## Introduction

Lumbar disc herniation (LDH) is a prevalent degenerative spinal condition, affecting approximately 77.8% of patients with lumbar spine disorders (1, 2). LDH is characterized by pathological displacement of disc material beyond the normal disc space. It typically occurs due to a combination of degenerative changes and mechanical stress. When disc herniation occurs, it may lead to compression of nearby nerve structures, resulting in various symptoms including lower back pain, numbness, and radiating pain to one or both lower limbs (3, 4). LDH is a major cause of discogenic low back pain and functional disability, significantly impacting patients’ quality of life and work capacity (5).

Self-management plays a crucial role in the prognosis of LDH, as it can significantly alleviate symptoms and improve functional outcomes. Studies have shown that structured self-management programs focusing on exercise, posture correction, and lifestyle modifications can lead to a reduction in pain intensity and enhance mobility in LDH patients (6, 7). Regular physical activity and targeted exercise programs not only reduce the frequency and severity of low back pain but also delay the progression of disc degeneration (8, 9). Additionally, self-management strategies empower patients to take an active role in their care, potentially improving adherence to treatment and rehabilitation protocols, which are essential for long-term recovery and quality of life (10). Given the high prevalence of recurrence and chronic disability associated with LDH, emphasizing patient education on self-management practices could be a key component in reducing healthcare utilization and promoting sustainable pain relief (11).

KAP surveys are effective diagnostic tools that assess individuals’ understanding, beliefs, and behaviors regarding specific health issues (12). In health literacy research, they are based on the premise that knowledge shapes attitudes, which then influence behaviors (13, 14). A lack of understanding of patients’ perspectives may lead to inadequate or misaligned care, highlighting the need for a patient-centered approach in LDH management. By examining KAP, healthcare providers can tailor educational interventions and self-management strategies to better meet the specific needs of LDH patients, potentially enhancing clinical outcomes.

To date, no studies have specifically focused on KAP in this patient group. This study aims to fill this gap by assessing the KAP of LDH patients regarding their condition.

## Materials and methods

### Study design and participants

This cross-sectional study was conducted from June to September 2024 at Tongren Hospital in Shanghai and the Seventh People’s Hospital of Changzhou, targeting patients with LDH. Ethical approval was granted by the Ethics Committee of Tongren Hospital, Shanghai (Approval No. K2023-016-01), and informed consent was obtained from all participants. The inclusion criteria were as follows: (1) patients with MRI or CT confirmed lumbar disc herniation (15); (2) individuals aged 18 years or older; (3) those capable of understanding the purpose of the study and willing to provide informed consent; and (4) participants who were fully conscious with basic cognitive function, allowing for comprehension and response to the questionnaire. The exclusion criteria included: (1) patients with severe comorbidities, such as advanced cancer or serious cardiopulmonary diseases; and (2) individuals with significant neurological disorders, spinal trauma, or other major conditions affecting the lumbar spine, such as spinal tumors, infections, or severe scoliosis with a Cobb angle greater than 60 degrees.

### Questionnaire

The questionnaire was developed based on previous literature (12) and relevant guidelines, including the *Guideline for the Diagnosis, Treatment, and Rehabilitation of Lumbar Disc Herniation* (16), and the *Clinical Practice Guideline for the Diagnosis and Treatment of Lumbar Disc Herniation* (15). After completing the initial draft, feedback was solicited from two experts with over 20 years of experience in spinal surgery. This input facilitated the enhancement of the baseline scale information and the refinement of the behavioral and practice sections. Subsequently, a preliminary survey involving 39 patients was conducted to assess the instrument’s reliability and validity. The overall Cronbach’s *α* coefficient was calculated to be 0.891, indicating strong internal consistency, with scores of 0.950, 0.761, and 0.833 for the knowledge, attitude, and practice sections, respectively. Throughout the pilot study, participants were encouraged to provide feedback on any items they found confusing or ambiguous; however, no such items were reported, thereby confirming the instrument’s face validity.

The final version of the questionnaire, which was administered in Chinese, comprised five sections: demographic data, knowledge, attitude, practice, and the Oswestry Disability Index (ODI) (Additional file 1. Questionnaire). The demographic section gathered information on variables such as age, gender, education level, residence, marital status, monthly income, duration of LDH, associated symptoms, surgical history, and living status.

The knowledge dimension included 11 items, scored as “very familiar” (2 points), “heard of it” (1 point), or “unclear” (0 points), with a total possible score ranging from 0 to 22 points. The attitude section consisted of 10 questions rated on a five-point Likert scale, ranging from “strongly agree” to “strongly disagree” (scored from 5 to 1), yielding a total score range of 10 to 50 points. The practice section included 12 items, with the final question regarding the information source about LDH (P4) used exclusively for descriptive analysis. The practice dimension was scored on a scale from “never” to “always” (1 to 5 points), yielding a total score range of 11 to 55 points. Furthermore, a common-sense trap question was incorporated to filter out invalid responses from participants who may not have reviewed the questions attentively. A threshold score of ≥70.0% of the total score was used to define adequate knowledge, positive attitudes, and proactive practices (17). The trap question, based on widely known information, stated, “Among China’s 56 ethnic groups, the Han ethnic group has the smallest population,” which is clearly false, as the Han is the largest ethnic group in China. This question was used to identify respondents who did not carefully review the questions, thereby invalidating their responses.

The ODI questionnaire provided a comprehensive assessment of the level of disability perceived by patients in various daily life activities (18). It consisted of 10 questions covering pain intensity, self-care, lifting, walking, sitting, standing, sleep interference, sexual activity, social life, and travel. Each question offered six response options, with scores ranging from 0 (no disability) to 5 (maximum disability), resulting in a total score range of 0 to 50, whereby higher scores indicated greater levels of disability.

### Questionnaire distribution and quality control

Both study hospitals are public, tertiary-care institutions located in economically developed coastal cities. The study was conducted in their routine orthopedic outpatient departments. All patients who attended the orthopedic outpatient clinic or were hospitalized with a confirmed diagnosis of LDH during the study period were consecutively approached by trained research staff. The study purpose was explained in detail, and patients were invited to participate voluntarily. After providing informed consent, patients completed the electronic questionnaire on-site or via their personal devices. The electronic questionnaire was distributed online via WeChat in orthopedic outpatient clinics and wards. Two designated research team members provided explanations of the questionnaire’s content and addressed any questions patients had while completing the survey. Participants who were unable or unwilling to use electronic devices were assisted by staff to ensure full participation.

### Sample size

The sample size was calculated using the formula for cross-sectional studies:


$$n={\left(\frac{z_{\left(1-\alpha /2\right)}}{\delta }\right)}^{2}\times p(1-p)$$

where Z_(1 − *α*/2)_ = 1.96 at a significance level of α = 0.05, p represents the anticipated proportion, and *δ* is the allowable margin of error (5% in this study). In the absence of prior data on LDH within this population, a conservative estimate of *p* = 0.5 was used, as this maximizes the required sample size. Based on a 95% confidence level and a 5% error margin, the calculated sample size was 384 participants. To account for potential attrition, an additional 20% was added, resulting in a final theoretical sample size of 480 participants.

### Statistical methods

Data analysis was performed using SPSS 26.0 (IBM, Armonk, NY, USA) and AMOS 24.0 (IBM, Armonk, NY, USA). The normality of score distributions across dimensions was assessed. The continuous variables conforming to the normal distribution were described as means ± standard deviations (SD) and analyzed using Student’s t-test (two groups) or ANOVA (more than two groups). The continuous variables with a skewed distribution were presented as median (P25, P75) and analyzed using the Wilcoxon-Mann–Whitney U-test or the Kruskal-Wallis analysis of variance. The categorical variables were described as n (%). Spearman’s correlation coefficient was used to evaluate relationships between dimensions. Structural equation modeling (SEM) was employed to assess whether attitudes mediated the relationship between knowledge and practice, as well as to compare the magnitude of direct and indirect effects. Goodness-of-fit criteria for SEM included the Root Mean Square Error of Approximation (RMSEA) < 0.08, the Standardized Root Mean Square Residual (SRMR) < 0.08, the Tucker-Lewis Index (TLI) > 0.80, and the Comparative Fit Index (CFI) > 0.80. A two-sided *p*-value of less than 0.05 was considered statistically significant.

## Results

### Demographic information on participants

Initially, a total of 468 questionnaires were collected. However, the following were excluded from the analysis: 10 participants who did not provide consent for the study, 2 individuals with response times of less than 90 s, 1 participant who was under 18 years of age, 2 participants with abnormal age values (self-reported age >180 years), and 58 respondents who answered the trap question incorrectly. Consequently, the final valid sample consisted of 395 questionnaires, resulting in an effective response rate of 84.40%. Among the respondents, 205 (51.90%) was female, 335 (84.81%) resided in urban areas, 149 (37.72%) held at least a bachelor’s degree, 184 (46.58%) reported having experienced LDH for fewer than 3 years, 201 (50.89%) had undergone surgical treatment, and 251 (64.54%) had received relevant education. Regarding symptom duration, 46.58% of patients reported having LDH symptoms for less than 3 years, while 53.42% experienced symptoms for 3 years or longer. Additionally, 270 patients (68.35%) reported complications suggestive of neurological involvement, including lower limb numbness (*n* = 162, 41.01%), muscle weakness (*n* = 72, 18.23%), gait disturbances (*n* = 34, 8.61%), and bowel and bladder dysfunction (*n* = 2, 0.51%).

The mean ± SD scores for knowledge, attitude, and practice were 14.91 ± 5.07 (possible range: 0–22), 39.26 ± 3.78 (possible range: 10–50), and 39.48 ± 6.70 (possible range: 11–55), respectively. These findings indicate inadequate knowledge, a positive attitude, and proactive practice when applying the cutoff value of 70% of the total score. The mean ± SD score for the ODI was 12.92 ± 9.60. Knowledge scores differed significantly by age (*p* = 0.004), residence (*p* < 0.001), education (*p* < 0.001), average monthly household income (*p* < 0.001), income type (*p* < 0.001), surgical treatment (*p* < 0.001), living status (*p* = 0.049), family history of LDH (*p* = 0.017), and relevant education (*p* < 0.001). Attitude scores varied significantly by average monthly household income (*p* = 0.009), surgical treatment (*p* < 0.001), and relevant education (*p* = 0.006). Practice scores were significantly different by residence (*p* = 0.009), comorbidities (*p* = 0.010), surgical treatment (*p* = 0.005), and relevant education (*p* = 0.005). ODI scores showed significant differences by age (*p* < 0.001), residence (*p* = 0.001), education (*p* < 0.001), average monthly household income (*p* < 0.001), marital status (*p* = 0.007), income type (*p* = 0.002), duration of LDH (*p* < 0.001), comorbidities (*p* < 0.001), and surgical treatment (*p* < 0.001) (Table 1).

**Table 1:** Baseline information and KAP scores.

Characteristics	N (%)	Knowledge, mean ± SD	*p*	Attitude, mean ± SD	*p*	Practice, mean ± SD	*p*	ODI, mean ± SD	*p*
*N* = 395		14.91 ± 5.07		39.26 ± 3.78		39.48 ± 6.70		12.92 ± 9.60	
Gender			0.489		0.499		0.142		0.209
Male	190 (48.10)	14.70 ± 5.12		39.16 ± 3.87		38.99 ± 6.60		12.39 ± 9.63	
Female	205 (51.90)	15.11 ± 5.03		39.35 ± 3.70		39.94 ± 6.78		13.41 ± 9.5816.50	
Age			0.004		0.079		0.676		<0.001
18–30	40 (10.13)	16.50 ± 4.72		39.58 ± 3.36		38.65 ± 7.51		8.40 ± 6.59	
31–40	69 (17.47)	16.19 ± 5.43		40.01 ± 4.37		38.94 ± 7.30		9.83 ± 9.13	
41–50	80 (20.25)	14.95 ± 5.22		39.30 ± 3.74		40.05 ± 6.14		12.23 ± 11.45	
51–60	59 (14.94)	14.63 ± 5.01		39.37 ± 3.93		38.97 ± 7.03		13.41 ± 8.61	
61–70	90 (22.78)	14.42 ± 4.64		38.77 ± 3.68		40.23 ± 6.49		14.64 ± 8.74	
>70	57 (14.43)	13.26 ± 4.91		38.72 ± 3.28		39.26 ± 6.16		17.58 ± 8.93	
Residence			<0.001		0.331		0.009		0.001
Rural	60 (15.19)	12.20 ± 4.75		38.73 ± 3.24		37.30 ± 6.71		16.35 ± 9.98	
Urban	335 (84.81)	15.40 ± 4.98		39.35 ± 3.86		39.87 ± 6.63		12.30 ± 9.42	
Education			<0.001		0.051		0.460		<0.001
Middle school or below	82 (20.76)	12.67 ± 4.47		38.62 ± 3.22		38.59 ± 6.88		16.10 ± 8.86	
High school/technical school	88 (22.28)	14.30 ± 4.93		39.09 ± 3.56		39.34 ± 6.95		15.13 ± 9.42	
Associate degree	76 (19.24)	15.04 ± 4.88		38.97 ± 4.12		39.54 ± 6.69		13.87 ± 10.27	
Bachelor’s degree and above	149 (37.72)	16.44 ± 5.09		39.85 ± 3.96		40.03 ± 6.46		9.38 ± 8.68	
Household’s average monthly income per person (RMB)			<0.001		0.009		0.470		<0.001
<5,000	91 (23.04)	13.46 ± 5.08		38.32 ± 3.29		39.53 ± 6.96		16.93 ± 9.49	
5,000–10,000	131 (33.16)	14.40 ± 4.83		39.04 ± 3.77		39.08 ± 6.45		12.27 ± 9.18	
10,000–20,000	89 (22.53)	15.98 ± 4.83		39.66 ± 3.43		39.08 ± 7.11		10.52 ± 8.76	
>20,000	84 (21.27)	16.15 ± 5.24		40.19 ± 4.38		40.49 ± 6.34		12.13 ± 10.07	
Marital status			0.413		0.815		0.229		0.007
Married	326 (82.53)	14.82 ± 5.10		39.27 ± 3.87		39.64 ± 6.67		13.55 ± 9.93	
Other	69 (17.47)	15.35 ± 4.96		39.22 ± 3.36		38.74 ± 6.83		9.94 ± 7.19	
Income type			<0.001		0.468		0.088		0.002
Stable income	350 (88.61)	15.31 ± 4.97		39.34 ± 3.81		39.67 ± 6.62		12.43 ± 9.50	
Unstable income	45 (11.39)	11.78 ± 4.82		38.64 ± 3.55		38.02 ± 7.22		16.71 ± 9.65	
Duration of LDH			0.156		0.374		0.558		<0.001
Less than 3 years	184 (46.58)	14.39 ± 5.30		39.33 ± 3.78		39.16 ± 7.05		10.33 ± 8.57	
3–9 years	125 (31.65)	15.18 ± 4.93		39.54 ± 3.53		39.80 ± 6.12		13.86 ± 9.94	
10–19 years	54 (13.67)	14.98 ± 4.97		38.63 ± 3.89		39.20 ± 6.28		15.37 ± 9.27	
20 years or more	32 (8.10)	16.72 ± 4.07		38.81 ± 4.52		40.53 ± 7.59		20.00 ± 9.49	
Comorbidities (lower limb numbness, weakness, gait disturbance, or bowel and bladder dysfunction)			0.575		0.324		0.010		<0.001
Yes	270 (68.35)	15.02 ± 5.08		39.43 ± 3.65		40.03 ± 6.52		15.02 ± 9.71	
No	125 (31.65)	14.67 ± 5.08		38.88 ± 4.03		38.30 ± 6.95		8.38 ± 7.61	
Surgical treatment			<0.001		<0.001		0.005		<0.001
Yes	201 (50.89)	15.74 ± 5.25		40.16 ± 3.70		40.30 ± 6.53		15.37 ± 10.13	
No	194 (49.11)	14.06 ± 4.75		38.32 ± 3.64		38.63 ± 6.79		10.38 ± 8.31	
Living alone			0.049		0.476		0.283		0.156
Yes	47 (11.90)	16.26 ± 5.04		39.40 ± 4.08		38.57 ± 6.59		11.02 ± 8.39	
No	348 (88.10)	14.73 ± 5.06		39.24 ± 3.74		39.60 ± 6.71		13.18 ± 9.74	
Family members with LDH			0.017		0.548		0.055		0.640
Yes	164 (41.52)	15.68 ± 4.96		39.39 ± 3.84		38.68 ± 6.76		13.41 ± 10.16	
No	231 (58.48)	14.37 ± 5.10		39.16 ± 3.74		40.05 ± 6.61		12.57 ± 9.20	
Medical educational on LDH			<0.001		0.006		<0.001		0.848
Yes	251 (64.54)	16.31 ± 4.54		39.68 ± 3.85		40.37 ± 6.46		13.02 ± 10.00	
No	144 (36.46)	12.47 ± 5.04		38.52 ± 3.55		37.92 ± 6.85		12.75 ± 8.89	

LDH, lumbar disc herniation.

### Knowledge, attitude, and practice

Regarding knowledge dimensions, the three questions most frequently answered as “Unclear” were: “Most patients with LDH have a good prognosis, with satisfactory treatment outcomes often achievable through conservative treatment, and potential for cure” (K10, 14.68%), “For patients whose pain severely impacts quality of life, analgesics like ibuprofen and diclofenac can be used” (K9, 13.92%), and “LDH is a syndrome resulting from degenerative changes in the lumbar disc, leading to nerve irritation or compression, and is a common clinical condition” (K1, 8.61%) (Table 2).

**Table 2:** Knowledge dimension.

Items	Very familiar, *n* (%)	Heard of it, *n* (%)	Unclear, *n* (%)	p*
Lumbar disc herniation (LDH) is a syndrome caused by degenerative changes in the lumbar disc, leading to nerve irritation or compression, and is a common clinical condition.	140 (35.44)	221 (55.95)	34 (8.61)	0.008
LDH commonly affects people who engage in heavy physical labor, frequently bend at the waist, or have sedentary jobs.	174 (44.05)	207 (52.41)	14 (3.54)	0.103
Most patients with LDH experience lower back pain and sciatica.	174 (44.05)	190 (48.1)	31 (7.85)	0.128
In severe cases of LDH, patients may experience numbness or weakness in the lower limbs, urinary or fecal dysfunction, or limping.	141 (35.7)	220 (55.7)	34 (8.61)	<0.001
LDH can only be diagnosed by combining clinical symptoms with imaging results (such as CT or MRI).	221 (55.95)	163 (41.27)	11 (2.78)	0.002
Treatment for LDH includes conservative treatment, medication, and surgery.	201 (50.89)	175 (44.3)	19 (4.81)	<0.001
For patients with mild symptoms and short disease duration, conservative treatment is preferred. If conservative treatment fails and symptoms are severe, surgery may be considered.	193 (48.86)	193 (48.86)	9 (2.28)	<0.001
Conservative treatment mainly includes lifestyle management, bed rest, appropriate activity, traction therapy, professional physiotherapy, acupuncture, and massage.	186 (47.09)	187 (47.34)	22 (5.57)	0.007
For patients whose pain severely affects their quality of life, analgesics can be used. Common drugs include ibuprofen and diclofenac.	148 (37.47)	192 (48.61)	55 (13.92)	0.022
Most patients with LDH have a good prognosis, and satisfactory treatment outcomes can usually be achieved through conservative treatment, with the possibility of cure.	116 (29.37)	221 (55.95)	58 (14.68)	0.003
LDH is a chronic progressive disease, and effective daily life management is important for treatment and preventing recurrence.	163 (41.27)	207 (52.41)	25 (6.33)	0.048

^*^Differences between patients with surgical treatment or not.

For attitudes, 10.89% disagreed that traditional Chinese medicine is more effective for treating LDH (A9), 9.37% were unwilling to undergo surgery even if recommended by a doctor (A7), and 7.59% were unwilling to use drugs for the condition (A6) (Table 3).

**Table 3:** Attitude dimension.

Items	Strongly agree, *n* (%)	Agree, *n* (%)	Neutral, *n* (%)	Disagree, *n* (%)	Strongly disagree, *n* (%)	p*
I believe that LDH is a serious disease.	82 (20.76)	192 (48.61)	91 (23.04)	29 (7.34)	1 (0.25)	0.004
I feel that LDH has affected my quality of life.	139 (35.19)	199 (50.38)	45 (11.39)	9 (2.28)	3 (0.76)	<0.001
I am confident in the effectiveness of treatment for LDH.	74 (18.73)	229 (57.97)	84 (21.27)	4 (1.01)	4 (1.01)	<0.001
I am willing to change my lifestyle according to the doctor’s recommendations.	132 (33.42)	247 (62.53)	15 (3.8)	/	1 (0.25)	<0.001
I believe that lifestyle changes can significantly improve the symptoms of LDH.	124 (31.39)	231 (58.48)	37 (9.37)	2 (0.51)	1 (0.25)	0.043
I am willing to use medication to treat LDH.	76 (19.24)	235 (59.49)	53 (13.42)	30 (7.59)	1 (0.25)	0.330
If recommended by a doctor, I am willing to undergo surgery to treat LDH.	74 (18.73)	195 (49.37)	83 (21.01)	37 (9.37)	6 (1.52)	<0.001
I am concerned about the risks and complications of lumbar surgery.	84 (21.27)	226 (57.22)	69 (17.47)	14 (3.54)	2 (0.51)	0.002
I believe that traditional Chinese medicine is more effective for treating LDH.	23 (5.82)	133 (33.67)	189 (47.85)	43 (10.89)	7 (1.77)	<0.001
I am willing to invest time and money to achieve better results for LDH.	78 (19.75)	237 (60)	64 (16.2)	13 (3.29)	3 (0.76)	<0.001

*Differences between patients with surgical treatment or not.

In terms of practice, 8.1% never used a lumbar support belt or back brace (P2), while 13.16% never underwent acupuncture, massage, or similar treatments (P3). For sources of information on LDH (P4), the most frequently reported sources were hospital-based publicity and education provided by supervising doctors or other healthcare professionals (80.76%), followed by new media platforms (63.54%) (Table 4).

**Table 4:** Practice dimension.

Items	Always, *n* (%)	Often, *n* (%)	Sometimes, *n* (%)	Rarely, *n* (%)	Never, *n* (%)	p*
I follow the doctor’s recommendation for regular check-ups.	88 (22.28)	162 (41.01)	117 (29.62)	21 (5.32)	7 (1.77)	0.003
How often do you follow these lifestyle habits?						
Regularly perform lumbar health exercises	38 (9.62)	149 (37.72)	137 (34.68)	66 (16.71)	5 (1.27)	0.015
Use a firm mattress	184 (46.58)	107 (27.09)	56 (14.18)	37 (9.37)	11 (2.78)	0.185
Use a lumbar support belt or back brace	47 (11.9)	102 (25.82)	143 (36.2)	71 (17.97)	32 (8.1)	0.060
Adjust your sitting or standing posture in daily life to avoid bad posture	85 (21.52)	163 (41.27)	119 (30.13)	26 (6.58)	2 (0.51)	0.029
Avoid prolonged standing or sitting	85 (21.52)	177 (44.81)	104 (26.33)	27 (6.84)	2 (0.51)	0.008
Keep your lower back and legs warm	103 (26.08)	183 (46.33)	87 (22.03)	20 (5.06)	2 (0.51)	0.001
Engage in moderate physical activity daily	64 (16.2)	133 (33.67)	144 (36.46)	50 (12.66)	4 (1.01)	0.329
Avoid intense physical exercise and heavy weight training	131 (33.16)	141 (35.7)	69 (17.47)	39 (9.87)	15 (3.8)	0.145
Follow a well-balanced diet and consume calcium-rich foods	71 (17.97)	169 (42.78)	126 (31.9)	25 (6.33)	4 (1.01)	0.017
I regularly undergo acupuncture, massage, or other similar treatments.	22 (5.57)	56 (14.18)	101 (25.57)	164 (41.52)	52 (13.16)	0.005

*Differences between patients with surgical treatment or not.

### Correlations between KAP

Correlation analysis revealed significant positive relationships between knowledge and attitude (*r* = 0.358, *p* < 0.001), as well as between knowledge and practice (*r* = 0.351, *p* < 0.001). Additionally, there was a significant correlation between attitude and practice (*r* = 0.367, *p* < 0.001) (Table 5).

**Table 5:** Correlation analysis.

Variables	Knowledge	Attitude	Practice
Knowledge	1		
Attitude	0.358 (*p* < 0.001)	1	
Practice	0.351 (*p* < 0.001)	0.367 (*p* < 0.001)	1

### Structural equation model

**Figure 1:**
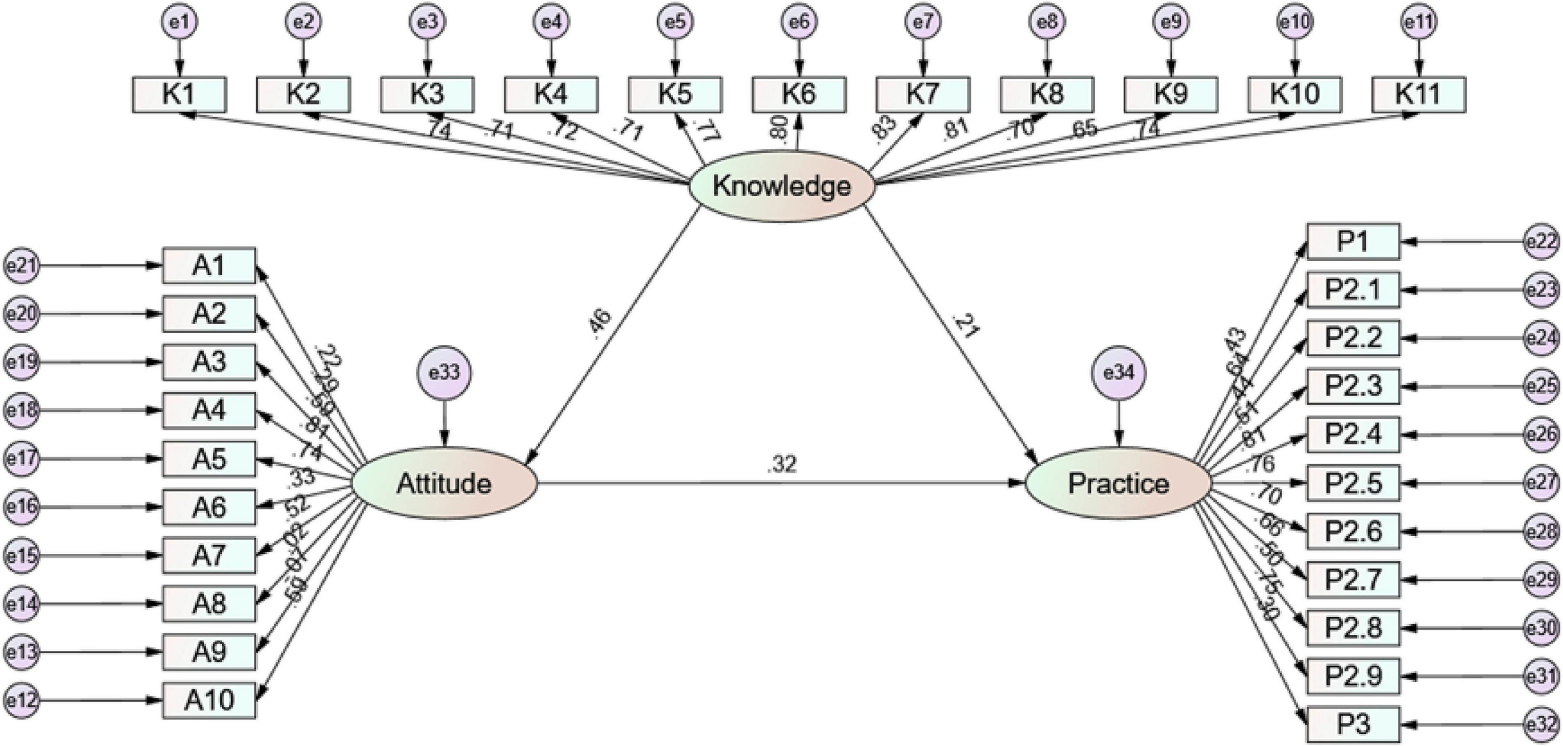
SEM analysis.

The SEM demonstrated good fit indices (CMIN/DF = 2.988, RMSEA = 0.071; IFI = 0.834; TLI = 0.820; CFI = 0.833) (Supplementary Table S1). Detailed path effects are presented in Supplementary Table S2. Analysis of direct and indirect effects indicated that knowledge had a direct effect on both attitude (*β* = 0.458, *p* = 0.006) and practice (*β* = 0.214, *p* = 0.002), while attitude directly affected practice (*β* = 0.323, *p* = 0.008). Knowledge also indirectly influenced practice via attitude (*β* = 0.148, p = 0.006) (Figure 1).

### Comparison between the surgical and the non-surgical patients

Furthermore, the comparison of KAP and ODI items between the surgical and the non-surgical patients were present in Tables 2–4 and Supplementary Table S3. ODI item analysis revealed that surgical patients reported significantly higher disability levels in most functional domains, particularly in lifting, walking, sitting, standing, sex life, social life, and traveling (all *p* < 0.001).

## Discussion

Patients with LDH demonstrated inadequate knowledge but maintained positive attitudes and proactive practices regarding their condition, with a moderate level of disability due to back pain.

This study’s findings on positive attitudes and proactive practices align with previous research on chronic pain conditions, where patients often demonstrate a strong willingness to pursue treatment options despite knowledge deficits (19, 20). However, the insufficient knowledge observed here parallels results from studies on conditions like osteoarthritis and chronic lower back pain, where limited understanding of the disease’s pathology and management has been linked to poorer self-care and delayed healthcare-seeking behavior (21, 22). The higher knowledge and practice scores observed among surgical patients suggest a possible association between perioperative education and improved understanding of the condition, though this finding may not be directly applicable to non-surgical patients. This suggests that the lack of knowledge could be a key factor contributing to less favorable outcomes in patients with LDH, as inadequate awareness of effective treatment options often results in prolonged symptoms or recurrence.

Regarding the relationships among KAP, both the correlation analyses and SEM results confirm significant direct effects of knowledge on attitudes and practices, with knowledge also indirectly influencing practices through attitudes. This is similar to findings in studies of other chronic conditions, where knowledge plays a central role in shaping attitudes, which in turn drive adherence to recommended practices (23, 24). These findings reinforce the importance of knowledge as a fundamental component of effective self-management strategies in chronic diseases, highlighting the potential benefits of targeted education interventions to enhance not only patient awareness but also practical health behaviors.

The study identified significant differences in knowledge scores based on age, residence, education level, and household income. The better knowledge among younger patients may be attributed to greater access to digital health information and engagement with new media platforms, which tend to be more popular among younger populations (25, 26). In contrast, older patients, particularly those over 60, had significantly lower knowledge scores. While this might be related to factors such as digital literacy or media preferences, further research would be needed to confirm these associations, emphasizing the need for age-appropriate educational materials that cater to their learning preferences.

Our results showed that urban residents displayed higher knowledge levels than rural residents. While this disparity might be related to factors such as healthcare access and exposure to health education (27, 28), future research would be valuable to understand the specific barriers faced by rural residents. Such understanding could help inform the development of targeted educational interventions.

In terms of education, patients with a bachelor’s degree or higher demonstrated significantly better knowledge and lower disability levels compared to those with less education. This disparity could be attributed to a higher likelihood of receiving relevant information through professional networks or educational channels (29, 30). Consequently, targeted educational programs should be developed for patients with lower educational backgrounds, using simplified language and visuals to enhance comprehension and engagement.

Surgical treatment appears to impact both KAP and ODI, with patients undergoing surgery reporting higher knowledge and practice scores but also increased disability levels. This may result from enhanced perioperative education by healthcare providers, improving patient understanding, and from the heightened awareness of physical limitations post-surgery. Additionally, as surgical intervention is often reserved for severe cases of LDH when conservative treatments are insufficient, this could also explain the observed higher ODI scores. The comparison between surgical and non-surgical patients yielded several important insights. Patients who had undergone surgical treatment generally demonstrated higher scores in knowledge and practice dimensions, as well as more favorable attitudes toward clinical interventions. This may be attributed to more intensive preoperative and postoperative education and closer physician-patient interaction during hospitalization. However, these patients also reported greater disability across most ODI domains. This might reflect the fact that surgical candidates often present with more severe baseline symptoms or functional impairment. The lack of difference in the “sleeping” domain may suggest that sleep quality is influenced by other factors beyond the scope of physical disability alone.

An analysis of the specific KAP items reveals that patients often lack confidence in the effectiveness of conservative treatments and pain management options, as many were uncertain about the prognosis and treatment outcomes. This finding is in line with previous studies, where low confidence in non-surgical treatments has been linked to delayed decision-making and a preference for surgical interventions (31, 32). The higher confidence and adherence to practices among surgical patients indicate that surgical intervention may reinforce patients’ beliefs in the effectiveness of medical guidance, potentially motivating them to engage in proactive health behaviors. Attitudinally, a significant proportion of patients expressed hesitancy toward traditional Chinese medicine and surgical treatments. Addressing these uncertainties through evidence-based information could help patients develop more balanced attitudes toward all available treatment options.

Some of the attitudes observed in this study, such as hesitancy toward surgery and skepticism about Western pharmacological treatments, may be influenced by cultural beliefs and traditional health practices in China. The relatively mixed views on TCM reflect the coexistence of TCM and modern medicine in China’s healthcare system, where patients often seek a balance between both approaches (33). Moreover, reluctance to undergo surgery may stem from cultural preferences for non-invasive therapies and fear of long-term consequences, which are common among East Asian populations. These cultural factors should be taken into account when interpreting patient attitudes, and they also highlight the importance of culturally sensitive education strategies in promoting evidence-based practices. Practice-wise, adherence to recommended lumbar support use and physical therapy techniques was lower than expected. This is consistent with findings in similar studies, which have reported that limited awareness and perceived ineffectiveness of these practices contribute to lower adherence rates (34, 35). Additionally, the relatively low uptake of acupuncture and massage therapy, despite their potential benefits, suggests a need for healthcare providers to discuss these options more effectively with patients, including clarifying the expected outcomes and appropriate use of these treatments.

To address the identified gaps in KAP, several targeted interventions are needed. First, hospital-based education programs should be tailored to enhance patient understanding of LDH. These programs could include interactive workshops that emphasize the nature, progression, and management of the condition, using simplified language, visual aids, and hands-on demonstrations, especially for older patients or those with lower education levels. Additionally, enhancing doctor-patient communication is crucial; healthcare providers should offer detailed explanations about treatment options, particularly emphasizing the effectiveness of conservative treatments. Addressing misconceptions and providing evidence-based information can help patients form realistic expectations and strengthen positive attitudes toward non-surgical interventions (36, 37).

For patients in rural areas, community outreach initiatives could be particularly effective. These programs could involve mobile health clinics that deliver educational materials, training, and demonstrations of lumbar support techniques, exercises, and other non-surgical interventions. Digital health resources, such as video tutorials and mobile apps, could further support patients by guiding them in performing lumbar exercises correctly, using lumbar support devices, and adopting lifestyle modifications. These digital tools should be designed with consideration for different age groups and literacy levels to ensure accessibility and effective learning (38, 39).

This study has several limitations. First, as a cross-sectional design, it cannot establish causal relationships between KAP among patients with LDH. Second, although all eligible patients were invited to participate, there may be selection bias, as those who consented could have higher health awareness, greater compliance, or more familiarity with digital tools, which may limit representativeness. Third, the reliance on self-reported questionnaires may introduce response bias due to recall inaccuracy or social desirability. Fourth, since the study was conducted in only two hospitals in economically developed regions of China, which may lead to an overrepresentation of patients with better access to medical resources and higher health literacy. As such, the findings may not be generalizable to patients in rural or underdeveloped regions. Additionally, 50.89% of the respondents had undergone surgical treatment, which is relatively high and could reflect the greater health-seeking behavior and compliance of surgical patients, possibly influencing the overall KAP scores. Lastly, although all included patients had imaging-confirmed LDH and self-reported symptoms, we did not stratify patients based on the type or severity of symptoms such as radicular pain, functional impairment or granularity of pain stratification. This may have influenced the interpretation of patients’ perceptions and responses regarding knowledge, attitudes, and practices. Future studies with larger, more diverse populations and detailed symptom stratification are warranted to validate and extend these findings.

## Conclusion

In conclusion, patients with LDH exhibited inadequate knowledge but generally positive attitudes and proactive practices toward managing their condition, with a moderate level of disability. The findings highlight that surgical interventions could enhance both patient knowledge and practice adherence, albeit with an associated increase in perceived disability. Enhancing patient education, particularly focusing on increasing knowledge about LDH, may further improve patient outcomes and self-management practices.

## Data Availability

The original contributions presented in the study are included in the article/Supplementary material, further inquiries can be directed to the corresponding authors.
