# Supplementary material for: Knowledge, attitudes, and practices regarding lumbar disc herniation among diagnosed patients
Source: Front Public Health. 2025 Jun 26;13:1583361. doi: 10.3389/fpubh.2025.1583361 (PMC12241107; doi:10.3389/fpubh.2025.1583361)
Supplement: Supplementary file 1 [file Table_1.docx]

# Table S1. SEM model fit

| **Model fit indices** | **Ref.** | **Measured results** |
| --- | --- | --- |
| **CMIN/DF** | 1-3 excellent，3-5 good | 2.988 |
| **RMSEA** | <0.08 good | 0.071 |
| **IFI** | >0.8 good | 0.834 |
| **TLI** | >0.8 good | 0.820 |
| **CFI** | >0.8 good | 0.833 |

Table S2. Analysis of direct and indirect effects

| **Model paths** | **Standardized Total effects** | | **Standardized direct effects** | | **Standardized indirect effects** | |
| --- | --- | --- | --- | --- | --- | --- |
|  | **β (95%CI)** | **P** | **β (95%CI)** | **P** | **β (95%CI)** | **P** |
| Knowledge → Attitude | 0.458 (0.364-0.562) | 0.006 | 0.458 (0.364-0.562) | 0.006 |  |  |
| Knowledge → Practice | 0.362 (0.268-0.458) | 0.004 | 0.214 (0.120-0.351) | 0.002 |  |  |
| Attitude → Practice | 0.323 (0.201-0.473) | 0.008 | 0.323 (0.201-0.473) | 0.008 |  |  |
| Knowledge → Practice |  |  |  |  | 0.148 (0.083-0.227) | 0.006 |

Table S3. The differences of ODI items between patients with surgical treatment or not

| ODI | P* |
| --- | --- |
| Pain intensity | 0.038 |
| Personal care (e.g. washing and dressing) | 0.021 |
| Lifting | <0.001 |
| Walking | <0.001 |
| Sitting | <0.001 |
| Standing | <0.001 |
| Sleeping | 0.451 |
| Sex life (if applicable) | <0.001 |
| Social life | <0.001 |
| Traveling | <0.001 |
